# The Therapist’s Intuition and Responsiveness: What Makes the Difference between Expert and in Training Gestalt Psychotherapists

**DOI:** 10.3390/ejihpe12120129

**Published:** 2022-12-07

**Authors:** Margherita Spagnuolo Lobb, Federica Sciacca, Serena Iacono Isidoro, Santo Di Nuovo

**Affiliations:** 1 Istituto di Gestalt HCC Human Communication Centre Italy, Via San Sebastiano 38, 96100 Siracusa, Italy; 2 Department of Educational Science, University of Catania, 95131 Catania, Italy; 3 Institute for Biomedical Research and Innovation (IRIB), National Research Council (CNR), 90146 Palermo, Italy

**Keywords:** aesthetic relational knowledge, Gestalt psychotherapy, psychotherapy training, therapist’s intuition, therapist’s responsiveness

## Abstract

This study aims to investigate the presence of intuition and responsiveness in early students and in experienced students and psychotherapists, which is understood as the ability to integrate bodily sensitivity and cognition of what is experienced with the patient (aesthetic relational knowing—ARK). The study compares how the therapist’s felt sense of the phenomenological intersubjective field and aesthetic relational competence differs between a group of experienced students and psychotherapists and a group of beginners. The sample consisted of 128 participants (20 M; 108 F), finally divided into two groups: “experienced students and psychotherapists” and “beginners”. The Aesthetic Relational Knowledge Scale (ARKS), a questionnaire consisting of 58 items targeting three factors (empathy, body awareness, and resonance), was administered. Statistical analyses were conducted to assess (i) differences between the two groups (through Student’s t and Cohen’s d for effect sizes), (ii) the influence of the level of training for each ARK factor using analyses of covariance for testing the possible influence of demographic variables, and (iii) logistic regressions to compare the influence of the three factors of the ARK model on the group variable with groups as a categorical variable. Significant differences between the two groups were found in body awareness and resonance. Body awareness was found to be the variable best discriminating between the beginners and the experienced students and psychotherapists. Despite being non-significant, there is a tendency suggesting that empathy appears more relevant at the beginning of training. The study shows the importance of training for the development of the therapist’s intuition and responsiveness, especially in the factors of body awareness and resonance. The results indicate the importance of assessing and supporting the aesthetic and field resonance of therapists in training, increasing quality and depth of the therapist’s responsiveness. This study is limited by a correlational design using self-report and on a limited sample, but it shows that the ARKS can monitor the effectiveness of training related to Gestalt therapeutic competencies.

## 1. Introduction

Over the past two decades, various studies [1,2,3,4,5] have explored the professional development of psychotherapists in training, both in terms of the intertwining of personal, professional, and method-related aspects and their impact on therapists’ empathic and intuitive skills. These investigations have important implications in order to better structure psychotherapy training [6,7,8]. Hill and Knox [9] highlighted the importance of tracking the predictors of the quality of psychotherapists’ training (e.g., curriculum, training components, personal therapy, and relational, personal, and perceptual characteristics).

This is a complex research perspective, due to the large number of variables that could be investigated [10,11], which needs to be focused on more specifically [12].

Much work on psychotherapists in training has focused on the effects of supervision during training and on a number of qualities that the therapist should have, including: skills and knowledge of concepts (e.g., academic knowledge, diagnostic skills, and understanding of parallel processes), developing the ability to conduct research [13], self-awareness (e.g., multicultural and countertransference), intrapersonal characteristics (e.g., self-efficacy and anxiety), specific skills (useful for helping and planning intervention), and self-assessment skills [11,12,14,15,16,17,18,19,20,21,22,23,24,25,26]. Recently, studies on psychotherapists’ relational skills and responsiveness have gained much interest [27], and in this study, we ask how much these skills are already present during training and how much they are influenced by it. Bennett-Levy [28] and Wampold [29] highlight that the most promising predictors of outcome include relational skills and professional development of psychotherapists in training [1]. The paradigm of responsiveness, that Stiles et al. [30] define as behaviors influenced by emerging events, such as therapist being influenced by and responding to what clients do, has been more recently described by Watson and Wiseman [27] (p. 3) as “a willingness or capacity to be flexible and fluid to align with and be attentive to their goals and needs”. In this study, we ask how much these skills are already present during training and how much they are influenced by it.

Although there are many studies on the relational skills that psychotherapists need to be effective, there are no studies that specifically indicate the therapist’s relational intuition and any development of it during training. The present study is concerned with the presence, in early-year students in psychotherapy and in more experienced students and psychotherapists, of a basic relational competence for therapist intuition, i.e., aesthetic relational knowledge (ARK). The term aesthetic refers to the original Greek term αἴσθησις (i.e., regarding sensory perception). The therapist’s relational intuition is based on the relational knowledge the ARK model provides, in a therapeutic relation and that can be explained as the ability to perceive, understand, and respond to the bodily processes and relational patterns enacted by the patient during the session.

In line with the need to study the predictive factors of responsiveness and therapeutic intuition, this study aims to investigate the presence during training of a specific and hitherto little-studied relational competence of the psychotherapist: the ability to integrate bodily sensitivity with cognition in what the therapist experiences of the phenomenological field that is created in the encounter with the patient. This aesthetic and relational knowing is a phenomenological expression of the therapist’s responsiveness [30]: a multidimensional construct that is part of therapeutic alliance studies [31,32,33,34,35,36,37,38] and of studies on reciprocity and synchronicity between psychotherapist and patient [37,38]. ARK, in fact, describes the therapist’s ability to grasp the patient’s movement towards and to establish a therapeutic relationship based on reciprocity and synchrony [39]. ARKS is the scale that measures this therapeutic ability [40].

### 1.1. The Construct of Aesthetic Relational Knowing (ARK)

ARK is defined as the intuitive “experience of the therapist that emerges from the phenomenological field created in a meeting between therapist and client” [40] (p. 10) and that “supports the therapist to understand the patient’s suffering in the field perspective, thanks to their isomorphic and aesthetic (i.e., based on sensorial perception) capacities” [40] (p. 3) [41].

In a previous work [40] (p. 12), we have found that ARK is described by three factors: empathy (the therapist’s ability to identify with the patient’s emotions), resonance (the therapist’s ability to experience “the other side of the moon” of the patient’s feeling, the contribution of the other to the co-created experience), and bodily awareness (“the therapist’s interoceptive ability to recognize the emotional-bodily activation in their own body”). 

The construct of ARK includes bodily awareness (in our previous work defined as “the ability to consciously perceive muscle tension, movement posture, heartbeat, breathing, satiety, and autonomic nervous system sensations related to emotions” [40] (p. 6) as a prerequisite for the therapist who uses their own perception as a resonance of the experiential field co-created with the patient [40]. 

Resonance and empathy are also key elements of the construct, which are closely related to each other. Resonance is, in our opinion, a different concept than empathy. It is the direct experience of temporarily entering into the perception, sensory, cognitive, and relational feeling of another [42,43,44,45]. “Resonance implies that feelings emerge within the entire intersubjective field of the therapeutic relationship and thus also the possibility of feeling what the significant other, with whom the experience has been co-created, feels in that situation” [39] (p. 59). 

Empathy, on the other hand, is the “ability to deliberately adopt the perspective of others” [40] (p. 4) and identify with their feelings. It has recently been described as consisting of three factors [46,47,48]: emotional contagion (feeling the same emotions that the other does), cognitive empathy (ability to understand and mentalize another’s feelings), and emotional disconnection (regulatory factor involving self-protection from distress and pain). In the ARK perspective, empathy is the therapist’s ability to identify with the patient’s emotions [40].

### 1.2. Aims

In this study, we aim to measure how the therapist’s felt sense of the phenomenological intersubjective field and aesthetic relational competence differs between a group of psychotherapists and experienced students and a group of beginners. In detail, we aim:

1. to explore how aesthetic relational knowing, a basic therapeutic skill, is present in novices and experienced students and psychotherapists;
2. to explore which dimensions of aesthetic relational knowing significantly differentiate levels of expertise in psychotherapy;
3. to advise training programs about which dimensions of psychotherapists’ intuition are less present at the beginning of training, and therefore to orient trainers about contents and modes to be supported in first year students.

## 2. Materials and Methods

### 2.1. Sample

Participants were provided by the Istitute of Gestalt Therapy HCC Italy, recognized by the Minister for the Universities to conduct post-graduate 4-year training (2000 h) in psychotherapy. The psychotherapy program includes 500 h of training each year (70 h specific theory; 50 h general theory; 100 h of supervision; 150 h of clinical laboratory; 130 h of clinical practice in mental health services).

The sample consisted of 128 participants including 20 males, 108 females, and 1 undeclared. The sample was initially made up of three groups: 42 licensed psychotherapists, 19 students in the third and fourth year of training, and 67 students attending the first and second year of training. 

A preliminary comparison was conducted to assess if there were any significant differences in the target variables between the trainees in the last years of training (third and fourth years, i.e., more than 750 h of supervised practice) and the already-trained psychotherapists. The analysis showed that there were no significant differences in the two groups in any of the three ARK factors (body awareness, resonance, and empathy). This allowed the creation of a unified group called “experienced students and psychotherapists”. 

Consequently, the sample for the analyses was composed of two groups. The first group consisted of 61 participants: 19 psychotherapy students attending the third and fourth year of training and 42 specialized psychotherapists. This group included 11 males and 50 females, with an average age of 39.88 years (SD = 8.44), of whom 3.3% had a medical degree and 96.7% had a psychology degree. This group was called “experienced students and psychotherapists”.

The second group of students attending the first and second year of training was called “beginners” and consisted of 67 participants including 9 males and 58 females, with a mean age of 33.11 (SD = 7.29), of whom 6% held a medical degree and 94% held a psychology degree. 

### 2.2. Procedures

Participants completed an online questionnaire between December 2021 and January 2022. They were asked to read and agree to the informed consent to participate in the research before starting the compilation. This study complies with the code of ethics approved by the Italian Association of Psychology (AIP) in 2015 and with Declaration of Helsinki. All participants have been fully informed that anonymity was assured, about the aims of the research, and how their data would be used (in aggregate form). The research adheres to the ICMJE guidelines.

### 2.3. Measures

Participants were administered the Aesthetic Relational Knowledge Scale (ARKS) [40], a questionnaire consisting of 58 items useful for assessing the therapist’s intuitive experience that emerges from the phenomenological field created in a therapist–patient encounter. The scale consists of the three factors described in the previous paragraph: empathy, body awareness, and resonance. Participants were asked to respond on a 7-point Likert response scale, ranging from 1 (extremely disagree) to 7 (extremely agree).

The reliability of the whole scale, based on Cronbach’s alpha, was 0.87. Reliability of body awareness was 0.921, reliability of empathy was 0.672, and reliability of resonance was 0.730.

Socio-demographic questions, such as gender, age, and education level, were also proposed to analyze the sample.

### 2.4. Data Analysis

Statistical analyses aimed at assessing (i) differences between groups (through Student’s t and Cohen’s d for effect sizes), (ii) the influence of the level of training for each ARK factor using analyses of covariance for testing the possible influence of demographic variables, and (iii) logistic regressions to compare the influence of the three factors of the ARK model on the group variable with groups as categorical variable.

## 3. Results

We have tested, by means of *t*-test for separate groups, the differences between “experienced students and psychotherapists” and “beginners” in the three factors of ARK. The results (Table 1) showed that there were significant differences between the two groups in the two factors of body awareness and resonance. In particular, “experienced students and psychotherapists” showed higher body awareness and resonance than “beginners”. In contrast, the two groups showed an inversed difference in empathy, although not statistically significant.

Next, the possible influence that variables such as gender, age, and level of training have on the three factors of ARK was tested, as shown in Table 2. In the table, the main effect is named “groups”, while other demographic variables (gender, considered as dummy variable, and age) are used as covariates. 

The results of the analysis of covariance show that *neither gender nor age influence the principal effect, in none of the variables*. Gender does not covariate with body awareness and empathy, while it is almost significant for resonance (*p* = 0.07). Age does not affect the main effect (more or less training of psychotherapists). 

Finally, a logistic regression was conducted to compare the influence of the three factors of the ARK model on the group variable (“experienced students and psychotherapists” vs. “beginners”), as shown in Table 3. 

The results showed that body awareness affects more than resonance in differentiating the two groups, while empathy seems to affect the experience in the opposite direction. Body awareness is confirmed to be the variable best discriminating between beginners and experienced students and psychotherapists, while empathy (as described in the ARK model) appears to be more relevant in the earliest stages of training. 

## 4. Discussion

As hypothesized in aim 1 of our study, results show that there is a meaningful difference in the ARK variables between “experienced students and therapists” (third- and fourth-year trainees and already-specialized therapists) and “beginners” (first and second year of training). Regarding aim 2 (to explore which dimensions of the aesthetic relational knowing significantly differentiate levels of expertise in psychotherapy), we have found that the meaningful difference between the two groups regards, in particular, two of the three factors of ARK: bodily awareness and resonance. We should remember that bodily awareness—described as “the therapist’s interoceptive ability to recognize the emotional-bodily activation in their own body” [40] (p. 12)—is the factor that shows the most significant difference between “experienced students and psychotherapists” and “beginners”. Resonance—described as the therapist’s ability to experience the contribution of the other to the (co-created) experience of the patient—shows also a significant, even if less meaningful, difference between first-year students and experienced students and psychotherapists. 

An interesting finding is that empathy does not show a meaningful difference in the two groups. Instead, in the present study, empathy—compared with other ARK factors—appears to be more relevant in less experienced students. This finding can be explained with the natural attitude of the psychotherapy students (some of them with years of experience in psychological counseling) to participate in the other’s emotions, while the structured training fosters the learning to “distance oneself” from the patients’ emotions in order to help them at a professional level as a psychotherapist. 

### Limits of the Study

Although the ARKS has been validated in a pilot study, and its psychometric properties appear to be satisfying, there is limited research using this instrument. ARKS has been developed only recently; it will be necessary to further validate the scale by administering it along with other measures that could support its construct validity. Moreover, we intend to correlate results from ARKS with clinical outcomes. 

The study is based on a correlational design using only self-report measures. Future research should use clinical interviews, and possibly data on clinical outcomes, to gather additional information.

Moreover, we have split participants into two groups (beginners and experienced students and psychotherapists) to check for meaningful differences, but in a future study, we could use, as continuous variables, the years of training in psychotherapy and of previous basic experience as a counselor, to have more detailed results not available with our present data. 

Another limitation of this first study is that the sample consists exclusively of Gestalt psychotherapists, due to the diffusion of ARK construct mainly within this model. It will be interesting to verify in subsequent research whether these findings are specific to the Gestalt therapeutic approach, or whether they are confirmed in other approaches as well.

Given that ARK is defined as “the capacity of psychotherapists to have a felt sense of the patient’s situation and contextualize it in a field/relational perspective”, we are dealing with a competence that is shared with other models in clinical practice, although described in specific languages. ARK expresses a meta competence that focuses on the bodily experience, both of the therapist and the patient, and the phenomenological field experienced by both in the here and now of the session, from which intentionality emerges that the therapists grasp with their aesthetic intuition. 

It will be even more interesting to measure this capacity in students and psychotherapists from other approaches. As we know, theoretical and methodological differences, that are evident when students learn a method, become less important when experienced psychotherapists dialogue and work together [48]. There is a shared implicit language that all psychotherapists speak that ARK could support.

## 5. Conclusions

“The results of research on psychotherapies drive the professional community to pay more attention to the quality of the training of therapists, based on integration of theoretical-epistemic and technical-methodological aspects” [13] (p. 328). The capacity of psychotherapists to have a felt sense of the patient’s situation and contextualize it in a field/relational perspective (a therapeutic competence that we call “aesthetic relational knowing” [40]) is definitely learnt after the training. The dimensions of this competence more sensitive to training are bodily awareness and resonance. Moreover, empathy, defined as the therapist’s ability to identify with the patient’s emotions, can be influenced by training, although in opposite direction, i.e., promoting more professional distancing from the patients’ emotions. Empathy is a natural attitude that belongs to students who choose to become psychotherapists, and who have likely gone through, in all their life, a reflective process about themselves, and the others, who have a spontaneous curiosity towards human relationships, their processes, functioning, and meanings. This spontaneous attitude to empathy of psychotherapy students needs to be carefully nurtured by trainers, since it might be more regulated (not depleted) during training experience. 

These findings have implications for training therapists and optimizing their therapeutic capacities, as we have advocated with aim 3 of this study: to advise training programs about which dimensions of psychotherapists’ intuition are more or less present at the beginning of training, and therefore orient trainers about contents and modes to be supported in first- and second-year students. 

Confirmation of the first purpose of this study leads us to emphasize the importance of fostering not only body awareness experiences, but also resonance experiences in trainees. Already, Pintado [49] had stated that the clinical psychologist about to become a psychotherapist must take care of himself or herself by developing self-awareness [50], understood as awareness of bodily, emotional, and mental aspects [51]. In this direction, studies conducted by Orlinsky and Rønnestad [1] and Rønnestad et al. [12] have highlighted the desirability of having trainees experience the healing power in the relational process to minimize any maladaptive experiences of stress. 

With respect to the development of empathy, some of our studies had shown that the desire to help, which emerges when one sees pain in the other, is linked with the ability to contain embodied empathy in a non-anxious way [52]. In another study [53], we have found that important relational skills, among which include empathy, are not influenced by the experience of training. On the other hand, Evers et al. [54] found that the personality trait “extroversion” is a predictor variable of therapist involvement and positive outcome of the therapeutic process, while the trait “neuroticism” predicts therapist distress. These spontaneous emotional dispositions have high relevance in the development of the therapist’s sensitivity to the phenomenological intersubjective field (resonance in ARK’s terms). 

It will be important to provide students of clinical specialization schools, besides theoretical frames, with personal experiences to be able to develop awareness of their body during the therapeutic session, and—a less developed competence—their capacity to use a field perspective [55,56] to deeply understand the mutual feelings that emerge in session (resonance). Additionally, therapists’ empathy should be regulated, within adequate professional boundaries. 

Moreover, we have seen that ARK, in its three dimensions, is not influenced by age and gender, while it can be influenced by training. The dimensions of bodily awareness and resonance are sensitive to change with the experience in spite of the students’ age and gender.

What our study specifically demonstrates is the importance of training for the development of the therapist’s intuition, relative to the two dimensions of body awareness and resonance. Psychotherapy training provides the ability to use one’s bodily feeling as an aesthetic tool to “feel/know” the patient(s) and their situation. In addition, the training provides the ability to locate this feeling of the therapist in the patient’s phenomenological field [39,57], in the complexity of their constitutive and current relationships. This is an important element of the quality and depth of the therapist’s intuition and responsiveness. 

The results indicate the importance for institutions providing training in psychotherapy to have procedures in place for assessing the aesthetic and field resonance of therapists in training and ensuring that therapists are provided with ongoing competent supervision. According to the study by Anderson et al. [58], assessment of these skills should already begin in the first year of psychotherapy training and could be achieved by administering or interviewing all those who begin this training. We can conclude that the use of ARKS, to describe the development of intuitive and responsive capacity of students, can be useful to monitor the efficacy of training related to these competences.

## Figures and Tables

**Table 1:** Differences between “beginners” and “experienced students and psychotherapists”.

		M	SD	t (df 126)	Cohen’s d	*p*-Value	95% Confidence Interval of the Difference	
							Lower	Upper
**Body awareness**	**Experienced students and psychotherapists**	**4.01**	**0.58**	**3.56**	**0.63**	<0.001	0.16	0.56
	Beginners	3.65	0.55					
**Resonance**	experienced students and psychotherapists	4.30	0.41	2.54	0.45	0.01	0.04	0.33
	Beginners	4.12	0.40					
**Empathy**	experienced students and psychotherapists	3.49	0.46	−1.05	-0.18	0.29	−0.25	0.08
	Beginners	3.58	0.46					

M—mean; SD—standard deviation.

**Table 2:** Analysis of covariance to assess the influence of gender and age on the differences in level of training for the three factors of ARK.

	Body Awareness		Resonance		Empathy	
	F-Ratio	*p*-Value	F-Ratio	*p*-Value	F-Ratio	*p*-Value
**groups**	12.44	<0.001	7.18	0.01	1.06	0.30
**gender**	0.04	0.84	3.41	0.07	0.09	0.77
**groups**	8.04	0.01	3.86	0.05	1.32	0.25
**age**	1.19	0.28	0.85	0.36	0.21	0.65

**Table 3:** Logistic regression of the three factors of ARK on variable “groups” (experienced students and psychotherapists vs. beginners).

	Estimate	Standard Error	Z	*p*-Value	Odds Ratio	Standard Error	95% Confidence Interval	
							Lower	Upper
**Constant**	**−3.80**	**2.21**	**−1.72**	**0.09**				
**Body awareness**	0.96	0.41	2.35	0.02	2.61	1.07	1.17	5.82
**Resonance**	0.57	0.57	0.99	0.32	1.76	1.01	0.57	5.42
**Empathy**	−0.67	0.43	−1.55	0.12	0.51	0.22	0.22	1.19

## Data Availability

Publicly available datasets were analyzed in this study. This data can be found here: https://docs.google.com/spreadsheets/d/1f8cuBaqUnzOEJTRtIpZ75k2S7N5I2np4tacgpdbJIKE/ (accessed on 25 October 2022).

## References

[1] Orlinsky D.E., Rønnestad H.M. (2005). The Collaborative Research Network of the Society for Psychotherapy Research. How Psychotherapists Develop: A Study of Therapeutic Work and Professional Growth.

[2] Orlinsky D.E., Strauss B., Rønnestad M.H., Hill C., Castonguay L., Willutzki U., Hartmann A., Taubner S., Carlsson J. (2015). A collaborative study of development in psychotherapy trainees. Psychother. Bull..

[3] Rønnestad M.H., Skovholt T.M. (2003). The journey of the counselor and therapist: Research findings and perspectives on professional development. J. Career Dev..

[4] Skovholt T.M., Jennings L. (2004). Master Therapists: Exploring Expertise in Therapy and Counselling.

[5] Skovholt T.M., Rønnestad M.H. (1995). The Evolving Professional Self: Stages and Themes in Therapist and Counsellor Development.

[6] Boswell J.F., Castonguay L.G. (2007). Psychotherapy training: Suggestions for core ingredients and future research. Psychother. Theory Res. Pract. Train..

[7] Fauth J., Gates S., Vinca M.A., Boles S., Hayes J.A. (2007). Big ideas for psychotherapy training. Psychother. Theory Res. Pract. Train..

[8] Klein R.H., Bernard H.S., Schermer V.L. (2011). On Becoming a Psychotherapist: The Personal and Professional Journey.

[9] Hill C.E., Knox S., Lambert M.J. (2013). Training and Supervision in Psychotherapy: Evidence for Effective Practice. Handbook of Psychotherapy and Behavior Change.

[10] Strauss B., Kohl S. (2009). Themen der ausbildungsforschung in der psychotherapie [Themes in research on training in psychotherapy]. Psychotherapeut.

[11] Carlsson J. (2012). Research on psychotherapists’ professional development during and after training. Nord. Psychol..

[12] Rønnestad M.H., Orlinsky D.E., Schröder T.A., Skovholt T.M., Willutzki U. (2019). The professional development of counsellors and psychotherapists: Implications of empirical studies for supervision, training and practice. Couns. Psychother. Res..

[13] Di Nuovo S. (2019). What research for what training in psychotherapy? Some methodological issues and a proposal. Res. Psychother. Psychopathol. Process Outcome.

[14] Rønnestad M.H., Ladany N. (2006). The impact of psychotherapy training: Introduction to the special section. Psychother. Res..

[15] Grater H.A. (1985). Stages in psychotherapy supervision: From therapy skills to skilled therapists. Prof. Psychol. Res. Pract..

[16] Hess A.K. (1987). Psychotherapy supervision: Stages, Buber, and a theory of relationship. Prof. Psychol. Res. Pract..

[17] Hill C.E., Castonguay L.G., Castonguay L.G., Hill C.E. (2017). Therapist Effects: Integration and Conclusion. How and Why Are Some Therapists Better Than Others? Understanding Therapist Effects.

[18] Hill C.E., Charles C., Reed K.G. (1981). A longitudinal analysis of changes in counseling skills during doctoral training in counseling psychology. J. Couns. Psychol..

[19] Jablon M. (1987). Psychotherapists’ perceptions of their professional development. Diss. Abstr. Int..

[20] Stoltenberg C. (1981). Approaching supervision from a developmental perspective: The counselor complexity model. J. Couns. Psychol..

[21] Stoltenberg C.D., Delworth U. (1987). Supervising Counsellors and Therapists: A Developmental Approach.

[22] Ladany N., Ellis M.V., Friedlander M.L. (1999). The supervisory working alliance, trainee self-efficacy, and satisfaction. J. Couns. Dev..

[23] Loganbill C., Hardy E., Delworth U. (1982). Supervision: A conceptual model. Couns. Psychol..

[24] Murphy D., Irfan N., Barnett H., Castledine E., Enescu L. (2018). A systematic review and meta-synthesis of qualitative research into mandatory personal psychotherapy during training. Couns. Psychother. Res..

[25] Ramos-Sánchez L., Esnil E., Goodwin A., Riggs S., Touster L.O., Wright L.K., Ratanasiripong P., Rodolfa E. (2002). Negative supervisory events: Effects on supervision and supervisory alliance. Prof. Psychol. Res. Pract..

[26] Watkins C.E. (2014). The supervisory alliance: A half century of theory, practice, and research in critical perspective. Am. J. Psychother..

[27] Watson J.C., Wiseman H. (2021). The Responsive Psychotherapist. Attuning to Clients in the Moment.

[28] Bennett-Levy J. (2019). Why therapists should walk the talk: The theoretical and empirical case for personal practice in therapist training and professional development. J. Behav. Ther. Exp. Psychiatry.

[29] Wampold B.E., Rousmaniere T.G., Goodyear R.K., Miller S.D., Wamplold B.E. (2017). What Should we Practice? A Contextual Model for How Psychotherapy Works. The Cycle of Excellence: Training, Supervision, and Deliberate Practice.

[30] Stiles W.B., Honos-Webb L., Surko M. (1998). Responsiveness in psychotherapy. Clin. Psychol. Sci. Pract..

[31] Heinonen E., Lindfors O., Laaksonen M.A., Knekt P. (2012). Therapists’ professional and personal characteristics as predictors of outcome in short- and long-term psychotherapy. J. Affect. Disord..

[32] Heinonen E., Lindfors O., Härkänen T., Virtala E., Jääskeläinen T., Knekt P. (2013). Therapists’ professional and personal characteristics as predictors of working alliance in short-term and long-term psychotherapies. Clin. Psychol. Psychother..

[33] Heinonen E., Knekt P., Jääskeläinen T., Lindfors O. (2014). Therapists’ professional and personal characteristics as predictors of outcome in long-term psychodynamic psychotherapy and psychoanalysis. Eur. Psychiatry.

[34] Nissen-Lie H.A., Monsen J.T., Rønnestad M.H. (2010). Therapist predictors of early patient-rated working alliance: A multilevel approach. Psychother. Res..

[35] Nissen-Lie H.A., Monsen J.T., Ulleberg P., Rønnestad M.H. (2013). Psychotherapists’ self-reports of their interpersonal functioning and difficulties in practice as predictors of patient outcome. Psychother. Res..

[36] Nissen-Lie H.A., Rønnestad M.H., Høglend P.A., Havik O.E., Solbakken O.A., Stiles T.C., Monsen J.T. (2017). Love yourself as a person, doubt yourself as a therapist?. Clin. Psychol. Psychother..

[37] Tschacher W., Rees G.M., Ramseyer F. (2014). Nonverbal synchrony and affect in dyadic interactions. Front. Psychol..

[38] Spagnuolo Lobb M. (2017). From losses of ego functions to the dance steps between psychotherapist and client. Phenomenology and aesthetics of contact in the psychotherapeutic field. Br. Gestalt J..

[39] Spagnuolo Lobb M. (2018). Aesthetic relational knowledge of the field: A revised concept of awareness in Gestalt therapy and contemporary psychiatry. Gestalt Rev..

[40] Spagnuolo Lobb M., Sciacca F., Iacono Isidoro S., Hichy Z. (2022). A measure for psychotherapist’s intuition: Construction, development, and pilot study of the Aesthetic Relational Knowledge Scale (ARKS). Humanist. Psychol..

[41] Spagnuolo Lobb M. (2016). Isomorphism: A Bridge to Connect Gestalt Therapy, Gestalt Theory and Neurosciences. Gestalt. Theory.

[42] Merleau-Ponty M. (1968). The Visible and the Invisible.

[43] Churchill S.D. (2007). Experiencing the other within the we: Phenomenology with a bonobo. Phenomenology.

[44] McConnell A.R. (2011). The multiple self-aspects framework: Self-concept representation and its implications. Personal. Soc. Psychol. Rev..

[45] Husserl E. (1965). Philosofie als Strenge Wissenschaft [Philosophy as a Rigorous Science].

[46] Decety J. (2011). The neuroevolution of empathy. Ann. N. Y. Acad. Sci. USA.

[47] Decety J., Michalska K.J. (2010). Neurodevelopmental changes in the circuits underlying empathy and sympathy from childhood to adulthood. Dev. Sci..

[48] Goldfried M.R. (2019). Obtaining consensus in psychotherapy: What holds us back?. Am. Psychol..

[49] Pintado S. (2019). Changes in body awareness and self-compassion in clinical psychology trainees through a mindfulness program. Complement. Ther. Clin. Pract..

[50] Baker E.K. (2003). Caring for Ourselves: A Therapist’s Guide to Personal and Professional Well-Being.

[51] Kinser P., Braun S., Deeb G., Carrico C., Dow A. (2016). “Awareness is the first step”: An interprofessional course on mindfulness & mindful-movement for healthcare professionals and students. Complement. Ther. Clin. Pract..

[52] Spagnuolo Lobb M., Sciacca F., Di Rosa A.R., Mazzone M. (2020). Bodily and Emotional Activation in Pain: Bridging Neurosciences and Gestalt Therapy to Understand the Therapist’s Wish for Help. Psychology.

[53] Alcaro A., Iacono Isidoro S., Conversi D., Accoto A., Spagnuolo Lobb M. (2020). The Emotional Personality of Psychotherapists: A Pilot Research with Gestalt-Therapy Clinicians. Psychology.

[54] Evers O., Schröder-Pfeifer P., Möller H., Taubner S. (2019). How do personal and professional characteristics influence the development of psychotherapists in training: Results from a longitudinal study. Res. Psychother. Psychopathol. Process Outcome.

[55] Spagnuolo Lobb M., Cavaleri P. (2023). Psychopathology of the Situation in Gestalt Therapy. A Field-Oriented Approach.

[56] Wollants G. (2012). Gestalt Therapy. Therapy of the Situation.

[57] Spagnuolo Lobb M. (2013). The Now-for-Next in Psychotherapy. Gestalt Therapy Recounted in Post-Modern Society.

[58] Anderson T., Crowley M.E.J., Himawan L., Holmberg J.K., Uhlin B.D. (2016). Therapist facilitative interpersonal skills and training status: A randomized clinical trial on alliance and outcome. Psychother. Res..

